# Contribution of traditional birth attendants to the formal health system in Ethiopia: the case of Afar region

**Published:** 2012-12-26

**Authors:** Tedla Mulatu Temesgen, Jemal Yousuf Umer, Dawit Seyoum Buda, Tilahun Nigatu Haregu

**Affiliations:** 1 AMREF, Ethiopia

**Keywords:** Traditional birth attendants, maternal and child health, pastoralist community

## Abstract

**Background:**

Traditional birth attendants (TBAs) have been a subject of discussion in the provision of maternal and newborn health care. The objective of this study was to assess the role of trained traditional birth attendants in maternal and newborn health care in Afar Regional State of Ethiopia.

**Methods:**

A qualitative study was used where 21 in-depth interviews and 6 focus group discussions were conducted with health service providers, trained traditional birth attendants, mothers, men, kebele leaders and district health personnel.

**Results:**

The findings of this study indicate that trained traditional birth attendants are the backbone of the maternal and child health development in pastoralist communities. However, the current numbers are inadequate and cannot meet the needs of the pastoralist communities including antenatal care, delivery, postnatal care and family planning. In addition to service delivery, all respondents agreed on multiple contributions of trained TBAs, which include counselling, child care, immunisation, postnatal care, detection of complication and other social services.

**Conclusion:**

Without deployment of adequate numbers of trained health workers for delivery services, trained traditional birth attendants remain vital for the rural community in need of maternal and child health care services. With close supportive supervision and evaluation of the trainings, the TBAs can greatly contribute to decreasing maternal and newborn mortality rates.

## Background

Ethiopia is Africa's second most populous country and one of the poorest. Geographically located in the Horn of Africa, the nation has an estimated population of 81 million. Of the total population, more than 84% live in the rural areas. Administratively, the country is divided into nine regional states and two cities. It is one of the sub-Saharan African countries with an unacceptably high maternal mortality ratio. Ethiopian Demographic Health Survey indicated that for the period 1998-2004 the maternal mortality ratio was 673 deaths per 100,000 live births with a range of 548-799 [1].

The national health policy of Ethiopia is rooted in the primary health care approach and is thus efficient in mobilising community resources. The implementation of the Health Extension Programme, which began in 2005, resulted in a significant improvement in the health sector. The Government of Ethiopia (GOE) has also established the Health Service Extension Programme (HSEP) which aims to improve equitable access to essential preventive and promotive health interventions and increase coverage to 85% of the population, by extending health services to communities through the deployment of 30,000 female health extension workers (HEW). The HSEP acknowledges the role of TBAs and calls for their incorporation into the system by serving as volunteers who work under the supervision of the HEWs.

The Afar Regional Health Bureau, which is accountable to the Regional Government, has done a lot to ensure that the national policies are beneficial to the pastoralist communities. Parts of the nation inhabited by pastoralist and semi-pastoralist communities have also benefited from such policies. However, poorly developed infrastructure and inadequate numbers of health workers is still hampering the full attainment of expected results.

With inadequate numbers of skilled health workers for service delivery, Trained Traditional Birth Attendants (TTBAs) remain vital contributors in ensuring provision of adequate maternal and child health care services in the rural areas [2, 3]. A recent survey by AMREF indicated that 92% of women in zone 3 of Afar regional state delivered their last child at home. Of these home deliveries, 90% of the women were attended to by a traditional birth attendant. This survey is consistent with the findings of the EDHS [1] which indicated that 95.8% of total deliveries in Afar were carried out at home.

In many African countries, training TBAs has been a key strategy to improving maternal and child health care. However, recent analyses have concluded that the impact of training TBAs on maternal mortality is low [4, 5]. An emphasis on large scale TBA training efforts could also be counterproductive, as it would hold back the training of the necessary numbers of medium level providers, particularly midwives. The main benefits of training TBAs appear to be improved referral and links with the formal health care system, but only where essential obstetric services are available [4, 5]. Some studies have observed that formal training is not a requirement for this function.

Studies have shown that people often prefer a TBA to a trained midwife, especially when the midwife is a young, unmarried girl without children. TBAs not only provide technical assistance, but also attend to and support the mother during the whole process of childbirth and thereafter. The work of TBAs is adapted and strictly bound to the social and cultural matrix to which they belong; their practices and beliefs are in accordance with the needs of the local community [5, 6].

More research is necessary to understand the contribution of TBAs to maternal health in the community. More studies are also needed to understand the level of collaboration, communication and co-operation between Trained Traditional Birth Attendants and formal health facilities. This study sought to fill this gap.

## Methods

### Study design

This was a qualitative study. The study used focus group discussions (FGDs) and individual in-depth interviews (IDIs) to collect data. A total of 21 IDIs and 6 FGDs were conducted with health service providers (HSPs), TTBAs, mothers, men, *kebele*leaders and district health managers across three zones of Afar Region.

### Study area

The study was conducted in Zone 1, Zone 3 and Zone 5 of Afar Regional State. Three districts (Gewane, Argoba and Awash) from Zone 3, two districts (Ayisaita and Dubti) from Zone 1 and one district (Telalak) from Zone 5 were selected for the study.

### Study population

The district health office (2) and head of women affairs (1) were interviewed about their views on TTBAs and whether they were willing to support the collaboration between TTBAs and formal health service providers. Women (3) from the community were also interviewed about their experiences with TTBAs during their pregnancy and delivery and about their perspective on maternity care in health facilities. The men (*kebele*leaders and married adult men) from the community were interviewed about their views on the role of TTBAs in the health system and their recommendations. Furthermore, 7 TTBAs were interviewed. Table 1 shows number and distribution of in-depth interview informants and FGDs (2 groups of TTBA, 2 groups of *kebele*leaders and 2 groups of mothers).

**Table 1:** The Study Population

District	IDI	FGD
**Awash**	1 DHO	
	1 HSP	
**Gewane**	2 TTBAs	1 group of TTBAs
	1 HSP	
	1 adult man	
**Dupti**	1 gynaecologist	
	1 MCH Nurse	
**Aysaita**	2 TTBAs	1 group of TTBAs
	1 adult man	1 group of *kebele* leaders
**Telalak**	2 TTBAs 1adult man	1 group of *kebele* leaders
	1 mother	1 group of mothers
**Argoba**	1 TTBA	1 group of mothers
	1 DHO, 1 DWA	
	2 mothers	
	1 adult man	

### Data collection

A total of 15 individuals participated in the field implementation, deployed as 5 teams of 3 persons. Each team consisted of 1 translator, 1 qualitative expert who acted as the moderator and 1 note taker. The team comprised public health, social science, midwifery and other medical professionals.

An in-depth interview (IDI) and focus group discussion (FGD) guide were developed with a participatory approach with all the team members. The individual IDIs and FGDs were tape-recorded after verbal consent was granted and finally translated and transcribed by data collectors.

Informants and participants were asked about four major issues related to the services provided by traditional birth attendants in their districts. They were asked about the contribution of TBAs to the health of the community, the support provided by the formal health system to facilitate and improve the contribution of TBAs and the relationship between TBAs and the formal health system. Approval for the study was obtained from Afar Regional Health Bureau. A letter of collaboration was issued from the Bureau and shared with the respondents. All participants were informed about the purpose of the study and the research methods that were being employed. Informed verbal consent was obtained from all respondents.

### Data analysis and interpretation

During data collection, field notes and audiotape recordings, as well as observations were carefully logged, transcribed by the trained qualitative experts, translated from the local language into English, coded and categorised. Codes were summarised to induct themes and analysed thematically with frequencies for interpretation interpreted accordingly. The findings were verified by members of the data collection team to ensure that the original discussions and interviews were well represented and not lost in the coding.

## Results

The key themes of the study are: TTBAs contribution, collaboration between TTBAs and the formal health system, the role of TTBAs in strengthening the referral linkage and challenges

### Contribution of TTBAs to maternal and child health

The respondents believed that TTBAs made a significant contribution to maternal and child health care and reproductive health. One respondent mentioned that TTBAs were highly respected in the community and women tell them all their secrets. A health service provider from Dubti hospital said, “TTBAs play a significant role in maternal health care since they decrease the workload of HSPs.”

### Family planning

The contribution of TTBAs in family planning is controversial. In some districts, FP is accepted and practised (e.g. Argoba), while in others, it remains a challenge (e.g. Awash). A TTBA in Argoba mentioned that everybody in her kebeleis interested in family planning, although most are against the permanent sterilisation method.

A TTBA in Gewane disclosed that she advises each woman that in order to remain strong and healthy, she has to “deliver the children with enough space in between”. Some interviewees mentioned that there has been some change in the communities due to the TTBAs’ increasing awareness of FP, although religion is still a major influence.

### Antenatal care

All of the respondents recognised the contribution of TTBAs in screening at-risk mothers during pregnancy. According to a health service provider in Gewane, TTBAs are trained in identifying danger signs associated with pregnancy. Besides the screening role and the contribution in encouragement of utilisation of ANC services at a health facility, all of the interviewees mentioned the counselling service that TTBAs provide as a significant support. They educate the pregnant women on the importance of personal and environmental hygiene, nutrition, immunisation, malaria prevention and healthy behaviour. There was consensus that TTBAs usually refer a woman to a health facility when she gains too much weight, when she is too tired, when she has abnormal oedema, hypertension, little foetal movement, blood loss, malaria and when she still vomits after six months.

### Delivery

The majority of the interviewees mentioned the TTBAs’ contribution towards delivery care. Most women prefer to give birth at home. A health service provider in Dupti confirmed this when he said, “We serve about 15% of pregnant women during ANC, but only 3% of these will eventually deliver in our hospital.” The TTBAs were in agreement that during delivery, they would refer a woman to the HF after abnormal presentation, prolonged labour, obstructed labour and excessive blood loss. Some TTBAs refer after four hours of labour. They learned during the training to refer patients early, in order to have adequate time to arrange for transportation and money. According to a HSP from Dubti, most cases of referral during delivery are obstructed and prolonged labour. The HSP from Dubti told of a case of two uteruses (abnormal uterus). A woman was referred to Dubti hospital after giving birth to a male neonate and its placenta at home. The delivery was attended by an elderly TTBA in the village. However, after the first delivery, the TTBA still felt an undelivered baby abdominally. Due to her experience of handling twin pregnancies, she knew that the placenta always comes after delivering the last baby. Thus, she referred the women to Dubti hospital. Ultrasound suggested two uteruses and two cervixes. A caesarean section was performed to rescue the second baby. The TTBA told the HSP that it was due to the training that she was able to recognise this danger sign.

### Child care

All the participants who took part in the IDIs mentioned that TTBAs clean the newborn after delivery and when the baby is awake and alert, they encourage the mothers to breastfeed. The study also revealed that there still exist unacceptable practices such as feeding the newborn a mixture of sugar and (boiled) water, sometimes milk and butter. TTBAs also educate mothers on how to care for their newborns. A District Health Officer in Awash said, “After the training, TTBAs improved child care infection prevention and advise mothers on proper cord-care.”

### Postnatal care

After delivery, the majority of the TTBA respondents recognized the need to wash the mother with water and soap. They also teach their clients about the importance of personal hygiene and eating a healthy diet. Afari culture recommends that mothers and babies stay in the house for 40 days. During this period, drinking of water and eating of solid foods is prohibited. The women can only leave the house for some morning-sun and to urinate.

### As a social capital for MCH

Participants of FGDs and IDIs acknowledged the role that TTBAs played as health educators at the community level. They trained the community about the problems associated with FGC. It was mentioned that although a TTBA provides a special service to women, every community member has a social obligation to collaborate with the TTBA when the need arises.

### In prevention of harmful practices

TTBAs are also reported to be useful in the fight against harmful traditional practices. After the training, they began promoting behaviour change among community members and other untrained birth attendants. The move to stop some harmful cultural practices would have been impossible without involvement of insiders. TTBAs have been very effective in this area since, to a large extent, women are the ones affected by Harmful Traditional Practices.

### Collaboration between TTBAs and the health system

Collaboration of stakeholders is an important factor in effective service delivery. Access and utilisation of reproductive health services are affected by the interaction between the formal and traditional health systems. The formal health system, as a result of its limited capability to reach all the communities, opted to train traditional health service providers. Moreover, given that many deliveries occur at home in Afar and the geographical distribution of pastoralist communities, the traditional service providers should be recognised.

The following short story was narrated by a TTBA:

*Three years ago I went to the health centre with a woman in labour. I referred her due to prolonged labour. At the centre, the HSP did the pelvic examination to measure the dilatation. He told the woman that her child would not be expected soon, so he left after preparing the room. But soon after his departure, I discovered the foetal head crowning. I quickly put on my gloves and delivered the baby. When the HSP heard the newborn cry, he returned to the delivery room and asked who delivered this baby, and I replied, “God did”*.

### TTBAs’ need for collaboration with other health care providers

Beyond the advice they provide during the prenatal and post-natal period, and during home delivery, TTBAs are also central in referring women to the health facilities. When a woman needs to be referred the TTBA will contact the husband to obtain permission and to accompany the patient to the hospital. The TTBA will also inform the adult men to arrange for transportation. Most communities use either the traditional wasaka or hire a car. Health systems would greatly improve if all community-based health agents were well co¬ordinated. There are mother co-ordinators working on malaria eradication, there are trained and untrained TBAs and a number of other health care agents. All these groups work on their own and hardly ever come together.

### Training

During the study, two types of training were mentioned: basic and refresher. Both were short term. There was no evidence of practical training or mentoring at the health facility level. The training was mainly conducted by NGOs, with limited participation by the district health management team. This definitely would have implications on sustainability of the operations. A District Health Officer in Gewane indicated, “It is challenging for the District Health Office to support them. There is no relationship between TTBAs and the District Health Office, so they don't come and ask for help. The District Health Office has very little information about what the TTBAs require since they only report to NGOs. I know that lack of supplies is a challenge for many of the birth attendants, but they rarely come to ask for assistance.”' Traditional birth attendants acknowledged the role of the health system and NGOs in training them. This was confirmed during all the seven interviews conducted with TTBAs. The provision of delivery kits following the training has also made the work of TTBAs easier. This was confirmed by FGD participants in Aysaita.

### Referral linkage

In this study, TTBAs indicated that when they have to refer cases the communication between them and the community is good. There are multiple channels and points of communication and referral which include referral from one TTBA to another and/or to a nearby health facility. Some of them prefer to first refer a woman to another TTBA. In this way they can discuss the case and decide together if further referral to a health facility is needed. When it is too difficult to arrange for referral, some TTBAs said that they try to get a health service provider to the home of the woman in labour. Relatives of the woman in labour will go to the health facility to ask for help. Most TTBAs said that the first line referral points are health posts (HP) close to them, and if the case is beyond the capacity of a HP the women have to be referred to other health facilities, like Awash Health Centre, Nazareth hospital or Dubti hospital.

### Supervision and reporting

In several instances TTBAs, HSPs and other interviewees indicated that as a result of weak linkages among the various systems, supervision and support from the formal health system is sporadic. In spite of this weakness, the study revealed that TTBAs can be helpful in the development of a community-based health system. For instance, a TTBA reporting to the Aysaita district health office indicated that in the three months prior to this study she had supported 25 deliveries.

### Challenges

TTBAs encountered several challenges while working. The training of TBAs is usually carried out by NGOs. Government agencies have not paid much attention to the activities of TBAs. Although coverage of the training is good, there has been little follow-up and support to the TTBAs. In most cases, they lack essential supplies such as scissors. In most cases, TBAs agree that the trainings are not long enough to address all the important issues. In Afar the workshops lasted 15-21 days only. The TTBAs mentioned that their services are not valued by the health facility workers. This is usually reflected by the way they are treated when they take mothers to the health facilities. Since the area is hot and arid, there is perennial scarcity of water. This has had a major impact on hygiene and sanitation. In addition, mothers to be referred to a health facility need to travel long distances of up to 80 km in some cases. Most of the health centres are understaffed and ill-equipped and therefore it is likely that on arrival at the health centre, the patient will receive upward referral to another facility.

## Discussion

The findings of this qualitative assessment on the role of TTBAs in the health care system support other studies that demonstrate the critical place of TTBAs in Africa and other developing countries [6, 7, 8]. As stated by a District Health Officer interviewed in Awash, these important community-recognized personnel make an immense contribution in the area of reproductive health in Afar. Probably one important factor that was reiterated by respondents was the fact that TBAs minimize health access barriers. In areas like Afar, where health facilities are distantly located, one would not be surprised that overwhelming support for TBAs exists. In such areas, communication networks such as roads are poor, rendering rural dwellers nearly helpless when labour sets on. The situation is much worse in the case of obstetric emergencies. It is these emergencies that contribute the greatest toll to maternal mortality. Since TBAs are trained to identify and offer emergency management where possible, they may be able to support efforts to improve maternal survival in Afar and other regions of Africa.

As reported in this study, TTBAs also play a great role in improving community health and in the fight against HIV/AIDS. TTBAs are a community-accepted group of people. Since they work in their local neighbourhoods, they have good understanding of the cultures that they belong to. Cultural barriers are known to be among the most important factors for successful penetration of positive health seeking behaviour practices [7, 8]. In this regard, improvement in health outcomes is more likely if the agents of communication and advocacy are community-owned persons such as TTBAs.

In Aysaita there was a case where a TTBA had just given birth and there was nobody in the community to help the other women in labour. The women had to rely on their neighbours to assist them. One neighbour who was not sure about what to do chose to take her client to the health centre. When they arrived, they did not receive any assistance, and the neighbour had to deliver the baby, despite her lack of skills in the area. This story narrated to the interviewer by a TTBA has several implications. First we can infer that TTBAs are essential in the attempts to reduce maternal and child mortality rates during delivery. Secondly, TTBAs need to have appropriate contact with the health workers, especially during those times when they cannot assist their clients, such as when they themselves are ill, have just had a baby or are away attending social occasions.

Some of the TTBA respondents in different districts mentioned that they received little recognition from health workers. At times when TTBAs came to the health facility along with patients, they were treated poorly. These sentiments underscore system resistance to the role of TTBAs also documented in similar studies [6, 9]. There is need to enhance cooperation and amiable referral mechanisms between health facilities and TTBAs, in order to improve health system effectiveness. Nevertheless, there are positive indications that cooperation may improve. For instance, co-operation between the health system and TTBAs is reflected through referral. A gynaecologist indicated that once pregnant mothers accompanied by the TTBAs came into the hospital, they were attended to accordingly. One male interviewee from Gewane mentioned that, since their training, TTBAs had started to advocate for the banning of HTPs.

In addition to the reports to the HFs most of the TTBAs also prepare reports to the woreda health offices, *kebele*leaders and NGOs. This means that these workers also feed into the health information management system. Such roles ensure that data is collected at community level. Since data collection is done by community-accepted persons, they are likely to collect more accurate data since TTBAs are trusted by the community. Thus TTBAs in Afar not only contribute to service provision but also make important contributions to other elements of the health care system.

## Conclusion

TTBAs remain a vital resource in rural Ethiopia, particularly in the provision of maternal and child health care services. With availability of requisite tools and equipment, close supportive supervision, access to continuing education and recognition by the formal health system, trained traditional birth attendants can effectively contribute towards efforts to decrease maternal and newborn mortality rates in the country. Both governmental and non-governmental bodies should give the necessary recognition and support to this cadre of traditional health service providers.
